# Are Drug Companies Living Up to Their Human Rights Responsibilities? The Perspective of the Former United Nations Special Rapporteur (2002-2008)

**DOI:** 10.1371/journal.pmed.1000330

**Published:** 2010-09-28

**Authors:** Paul Hunt, Rajat Khosla

**Affiliations:** 1 Human Rights Centre, Department of Law, University of Essex, Essex, United Kingdom; 2 Human Rights Centre, University of Essex, Essex, United Kingdom

## Abstract

The human rights responsibilities of drug companies have been considered for years by nongovernmental organizations, but were most sharply defined in a report by the UN Special Rapporteur on the right to health, submitted to the United Nations General Assembly in August 2008. The “Human Rights Guidelines for Pharmaceutical Companies in relation to Access to Medicines” include responsibilities for transparency, management, monitoring and accountability, pricing, and ethical marketing, and against lobbying for more protection in intellectual property laws, applying for patents for trivial modifications of existing medicines, inappropriate drug promotion, and excessive pricing. Two years after the release of the Guidelines, the *PLoS Medicine* Debate asks whether drug companies are living up to their human rights responsibilities. Sofia Gruskin and Zyde Raad from the Harvard School of Public Health say more assessment is needed of such responsibilities; Geralyn Ritter, Vice President of Global Public Policy and Corporate Responsibility at Merck & Co. argues that multiple stakeholders could do more to help States deliver the right to health; and Paul Hunt and Rajat Khosla introduce Mr. Hunt′s work as the UN Special Rapporteur on the right to the highest attainable standard of health, regarding the human rights responsibilities of pharmaceutical companies and access to medicines.

## Abstract 2

As one viewpoint of three in the *PLoS Medicine* Debate on whether drug companies are living up to their human rights responsibilities, Paul Hunt (the UN Special Rapporteur on the Right to Health) and Rajat Khosla argue that pharmaceutical companies have been noncompliant and that better accountability mechanisms are needed.

*This is the third of three viewpoints examining the question of whether pharmaceutical companies are living up to their human rights responsibilities.*

## Human Rights Guidelines for Pharmaceutical Companies

In 2008, one of us (PH), serving as the UN Special Rapporteur on the right to the highest attainable standard of health, submitted a report to the UN General Assembly titled *Human Rights Guidelines for Pharmaceutical Companies* *in Relation to Access to Medicines* [1]. The *Guidelines* include a preamble, 47 guidelines, and a commentary. All are based on the human rights principles set out in the Universal Declaration of Human Rights, the international right to the highest attainable standard of health codified in numerous national constitutions and human rights treaties, and other widely accepted standards such as some of those adopted by the World Health Organisation (WHO) [2].

Emerging from a long process of research and consultation with pharmaceutical companies and others—a process outlined in the UN report of 2008—the *Guidelines* recognise some important realities. For example: governments have the primary responsibility for increasing access to medicines; many health systems are failing, constituting a grave obstacle to increasing access to medicines; the pharmaceutical sector helps to save lives and reduce suffering; and pharmaceutical companies have a responsibility to enhance shareholder value.

But the *Guidelines* also reflect another equally important reality: pharmaceutical companies have human rights responsibilities. Critically, the *Guidelines* give content to these responsibilities, with two vital implications. First, the *Guidelines* can and should help to shape corporate policies, and second, they provide right-to-health standards in relation to which companies can and should be held accountable. The 47 guidelines are grouped in themes (Box 1 contains examples).

**Box 1. Human Rights Guidelines for Pharmaceutical Companies in Relation to Access to Medicines**

Based on the human rights principles set out in the Universal Declaration of Human Rights, the international right to the highest attainable standard of health codified in numerous national constitutions and human rights treaties, and other widely accepted standards, the 47 guidelines are organised into themes. Some highlights:

- Guideline 5, which requires companies to give “particular attention to the very poorest in all markets,” arises from the human rights of equality and nondiscrimination.
- Guidelines 6–8, which include the rebuttable presumption “in favour of the disclosure of information, held by the company, which relates to access to medicines” is based upon the human rights principle of transparency.
- Other guidelines apply transparency to the theme of public policy influence, advocacy, and lobbying (guidelines 17–19).
- Guidelines 26–32 address patents and licensing, including the vital role of commercial and noncommercial voluntary licences.
- Guidelines 33–37 address pricing, such as differential pricing between and within countries.
- Other themes include management, monitoring, and accountability; neglected diseases; corruption; clinical trials; ethical promotion and marketing; and public–private partnerships.

## Pharmaceutical Patents and the Right to Health

In June 2008, PH made a formal UN right-to-health mission to GlaxoSmithKline (GSK) that culminated in a report on the company's policies regarding access to medicines [3]. Although distinct, the two projects—the *Guidelines* and the GSK report—informed each other.

While the *Guidelines* apply to innovator, generic, and biotechnology companies, the GSK report had a particular preoccupation with the right-to-health duties of innovator (i.e., patent-holding) companies. The report emphasises that a pharmaceutical company, having developed a life-saving medicine, has performed a vitally important medical, public health, and right-to-health function. The “reward” for fulfilling this critically important social function is the grant of a patent—a limited monopoly—over the relevant medicine, enabling the company to make a profit, enhance shareholder value, and invest in further research and development. However, the company is granted a patent under express and implied terms. Society has legitimate expectations of a company holding the patent on a life-saving medicine. On the basis of the goals of dignity and well-being of individuals and communities, as well as of globally recognised standards, the right to health clarifies, and gives substance to, these terms and expectations. The agreement between society and the patent holder of a life-saving medicine grants privileges to, and places responsibilities on, the patent holder (Box 2).

**Box 2. Right-to-Health Responsibilities of Patent Holders of Life-Saving Medicines**

- The seminal right-to-health responsibility is to take all reasonable steps to make the medicine as accessible as possible, as soon as possible, to all those in need, within a viable business model.
- For example, as soon as the new medicine is marketed at higher prices (usually in high-income countries), the patent holder has a right-to-health responsibility to put in place a range of mechanisms, such as differential pricing between and within countries, to enhance access for all those who cannot afford those prices. Also, the patent holder has a right-to-health responsibility to develop formulations for children, the elderly, pregnant and lactating women, and extremes of climate.
- The agreement with society places a responsibility on the patent holder to take these steps, expeditiously and effectively, by way of deliberate, concrete, and targeted measures.
- If the patent is worked without these steps being taken, the patent holder is in breach of its right-to-health responsibilities.
- The success of a patent holder's actions will sometimes depend upon states, donors. and others fulfilling their responsibilities. Nonetheless, the patent holder has a right-to-health responsibility to do what it can.

When the right-to-health report on GSK was presented to the UN Human Rights Council in 2009, the UK representative commended the company for its full engagement with the Special Rapporteur and continued: “While States bear responsibility for ensuring that human rights are protected within their jurisdiction, businesses should also ensure that they conduct their activities in a manner that is consistent with enjoyment of human rights. The Special Rapporteur rightly notes that progressively achieving access to medicines for all who need them is an objective to which both state and non-state actors can and should contribute.” The UK delegate concluded: “We agree that pharmaceutical companies should support objective reporting on their access to medicines commitments. We encourage them to develop approaches, such as external validation, to support this” [4].

## Are Pharmaceutical Companies Living up to Their Human Rights Obligations?

In an editorial headed “Right-to-health responsibilities of pharmaceutical companies,” the editors of *The Lancet* observed in 2008 that the GSK report sets out “with reasonable precision how the right to health, in the international code of human rights, applies to the pharmaceutical industry.” The editorial went on to say that “Pharmaceutical companies help deliver the right to health. They save lives. But with this role comes responsibilities – and companies must be better held to account in relation to those responsibilities. The 2008 guidelines and the GSK report move us closer to that goal” [5].

While many pharmaceutical companies are making an indispensable contribution to improving access to medicines and realising the right to health, they are not living up to all their human rights responsibilities. In 2008, and again in 2010, the Access to Medicine Index compared the efforts of 20 drug companies in relation to access to medicines [6]. The Index scored and ranked the companies on criteria such as public policy influence and advocacy, pricing, patents and licensing, and donation programmes. In 2008, GSK came out on top, followed by NovoNordisk, Merck & Co., and Novartis. The bottom-ranked companies were Pfizer, Wyeth, Teva Pharmaceutical, and Schering-Plough. In 2010, Gilead Sciences replaced NovoNordisk in the top four, and the bottom-ranked companies were Merck KGaA, Takeda Pharmaceutical, Astellas Pharma, and Daiichi Sankyo Co.

Although it does not explicitly adopt a human rights framework, the Index reflects aspects of the *Guidelines* and the right to the highest attainable standard of health, highlighting where companies are doing well in addition to some of their shortcomings. In effect, the Index signals the degree to which the selected companies are living up to some of their right-to-health obligations and which have progressed and regressed. It shows that the record is very mixed. As Wim Leereveld, the Index's founder, put it when launching the 2010 Index, “the industry as a whole still has a long way to go.”

The Special Rapporteur's GSK report recognises some of the company's achievements, but also identifies some deficiencies. For example, the report observes that while GSK deserves credit for significantly reducing some of its prices, thereby enhancing access, the price of Cervarix (HPV vaccine) “remains a cause of deep concern” (para 66). The report notes that “GSK's use of commercial and non-commercial voluntary licences has significantly enhanced access to some medicines,” observes that “GSK is not using voluntary licensing enough,” and concludes it is “critically important that GSK enters into more commercial and non-commercial voluntary licences across a range of medicines and markets” (paras 78, 79, 81). The report recognises that although “GSK is not usually considered to be an industry hardliner on intellectual property issues, some of its positions, such as those in India, Thailand and Philippines, undermine its leadership position”; thus the Special Rapporteur “urges GSK to respect the right of countries to use, to the full, TRIPS [Trade-Related aspects of Intellectual Property Rights] flexibilities and encourages GSK to make a public commitment not to lobby for TRIPS ‘plus’ standards” (para 82). The report commends GSK's appointment of an independent third party to externally assure information in the access to medicines section of the company's *Corporate Responsibility Report* (2007), but “greatly regrets” the failure to subject its next *Corporate Responsibility Report* to external assurance (para 102).

The publications of UK's Department for International Development, Pharma Futures, the George Institute for International Health, Oxfam, WHO, Health Action International, Access to Medicines Project, and others also tend to suggest that pharmaceutical companies are not living up to their human rights responsibilities [7]–[12]. Overall, we are left with the impression that insufficiently differentiated prices between and within countries, inadequate attention to research and development on some neglected diseases, incomplete disclosure of financial support for political candidates, inappropriate drug promotion, and problematic clinical trials, are all commonplace within the pharmaceutical sector. Crucially, they all represent a failure of pharmaceutical companies to comply with the human rights responsibilities set out in the *Guidelines* and the GSK report.

## The Next Step: Effective Right-to-Health Accountability Mechanisms

The apparent noncompliance of pharmaceutical companies with their human rights responsibilities underscores the vital importance of accountability mechanisms. Such mechanisms can serve to check whether or not these allegations are well founded and can make public, balanced, sensible determinations with practical recommendations for all parties. Both the *Guidelines* and the GSK report highlight the critical role of internal and external (i.e., independent) mechanisms that not only monitor companies, but also hold companies accountable for their right-to-health responsibilities. If a company is serious about its responsibilities to society, why not establish, for example, a corporate Ombuds with oversight of its right-to-health responsibilities relating to access to medicines? If courts are unable or unwilling to play this role, and if neither states nor companies have the appetite to establish effective right-to-health accountability mechanisms, then civil society must take the initiative, as it always has in the implementation of human rights.

In short, if others fail to act, a consortium of civil society organisations should appoint a panel of well-respected global leaders, supported by a small but properly resourced secretariat, to monitor the policies and practices of pharmaceutical companies and hold them publicly accountable for the discharge of their right-to-health responsibilities.

Today, we have the right-to-health standards to which pharmaceutical companies can be held accountable. The Access to Medicine Index and other publications provide a firm information base for monitoring the conduct of pharmaceutical companies [6]–[12]. But, while standards and monitoring are necessary, they are not sufficient. Now the challenge is to devise effective, fair, and appropriate mechanisms by which companies can be held publicly accountable for their right-to-health responsibilities.

## Abbreviations

GSK: GlaxoSmithKline
TRIPS: Trade-Related aspects of Intellectual Property Rights
